# Preoperative atrial fibrillation predicts worse outcomes after LVAD implantation

**DOI:** 10.34172/jcvtr.2022.29

**Published:** 2022-08-30

**Authors:** Moritz Benjamin Immohr, Yukiharu Sugimura, Esma Yilmaz, Hug Aubin, Udo Boeken, Payam Akhyari, Artur Lichtenberg, Hannan Dalyanoglu

**Affiliations:** Department of Cardiac Surgery, University Hospital of Düsseldorf, Düsseldorf, Germany

**Keywords:** Heart Failure, Vascular Complications, Left Ventricular Assist Device, Atrial Fibrillation, Extracorporeal Life Support

## Abstract

***Introduction:*** Left ventricular assist device (LVAD) implantation is a common therapy for end-stage heart failure. Heart failure patients often present with atrial fibrillation (AF). The purpose of this study was to evaluate the influence of preoperative AF as well as vascular complications on outcome in LVAD patients.

***Methods:*** Between 01/2010 and 12/2017, 168 patients (141 male) with end-stage heart failure underwent LVAD implantation at a single center. Patient outcome was retrospectively studied by using the Kaplan-Meier method for analyzing crude survival as well as Cox regression for analyzing risk factors.

***Results:*** Sixty-two patients suffered from preoperative atrial fibrillation at LVAD implantation. Mean age was 56.8±11.9 years (range: 22–79) and 141 (84%) were male. Postoperative vascular or visceral surgical management due to malperfusion was needed in 27 patients (16.1%) and did not correlate with postoperative mortality (*P*=0.121, HR=1.587, CI=0.885–2.845). Patients with preoperative AF had a worse outcome in the Kaplan-Meier analysis (*P*=0.069). In contrast, cox regression showed that postoperative AF could not to be considered to be an independent predictor of mortality in this study group.

***Conclusion:*** Our data suggest that preoperative AF may be a potential predictor of mortality and impaired long-term outcome in LVAD patients. In contrast, preoperative ECLS and vascular or visceral surgery after LVAD implantation did not represent limiting factors with regard to mortality after LVAD implantation.

## Introduction

 Atrial fibrillation (AF) and heart failure (HF) often coexist,^1^ and as many as nearly 30% of HF-patients are affected by AF.^2^ A common therapy for end-stage heart failure is the implantation of a permanent circulatory assist device, such as a left ventricular assist device (LVAD). Due to limited donor availability, the number of LVAD implantations has risen in the last decade, as has the number of patients who presented with preexisting AF. Compared with data on ventricular arrhythmia and sudden cardiac death, less is known about the incidence and impact of atrial arrhythmias in patients with LVADs. Unsurprisingly, the prevalence of preexisting atrial arrhythmias, particularly atrial fibrillation (AF), and the incidence of post-LVAD atrial arrhythmias is high.^3,4^ Atrial arrhythmias (AAs) are diagnosed in 21–54% of patients before LVAD implantation, most of these are AF and the rest are atrial flutter or atrial tachycardia.^5-9^

 Not only is AF considered to be a significant risk factor for stroke and death in general, it is also a significant risk factor for thromboembolic events after LVAD implantation.^10,11,12^ LVAD-related thromboembolic (TE) events, such as transient ischemic attack (TIA), stroke, and pump thrombosis, and non-thromboembolic events such as gastrointestinal (GI) bleeding have a devastating impact and have been extensively studied.^10,12,13,14^ However, the effect of AF in combination with malperfusion events (MPE) such as visceral ischemia, acute leg ischemia, or ischemia of the upper extremity on outcome in LVAD patients has not been well studied and is therefore poorly understood.

 The purpose of this study was to evaluate the influence of preoperative AF in combination with vascular complications on the outcome in LVAD patients.

## Materials and Methods

 Between 01/2010 and 12/2017, all patients with end-stage heart failure who were planned for LVAD implantation underwent a defined screening protocol. Data on LVAD implantation and the clinical course was retrieved from an institutional database. For the purposes of this study, data was retrospectively analyzed. The institutional review board (reference #2020 - 832) approved the retrospective data analysis. Informed consent had to be waived because of the retrospective nature of the study. Demographic and clinical characteristics of the study patients are presented in Table 1.

**Table 1:** Preoperative base line parameters of all included patients (n = 168) before LVAD.

**Variable **	**n (%)**	***p*** ** value **	**Hazard ratio for death (95.0% CI, lower – upper)**
Age > 65 years	40 (23.8)	0.282	1,310 (0.800 – 2.147)
Female gender	27 (16)	0.730	0.888 (0.753 – 1.741)
BMI > 30	30 (16.4)	0.137	1.498 (0.880 – 2.551)
Dialysis	11 (6.5)	0.259	1.568 (0.719 – 3.420
Nicotine	43 (25.6)	0.152	0.660 (0.374 – 1.165)
Diabetes mellitus	49 (29.2)	0.839	1.053 (0.641 – 1.729)
Atrial fibrillation	62 (36.9)	0.071	1.526 (0.964– 2.416)
COPD (chronic obstructive pulmonary disease)	28 (16.7)	0.123	1.550 (0.889 – 2.704)
CVD (cerebrovascular disease)	8 (4.8)	0.040	2.402 (1.040 – 5.552
Peripheral arterial disease	15 (8.9)	0.739	0.876 (0.401 – 1.910)
Angina pectoris	154 (91.7)	0.016	0.439 (0.224 – 0.860)
Syncope	12 (7.1)	0.284	1.532 (0.702 – 3.342)
Cardiogenic shock	108 (64.3)	0.914	1.027 (0.637 – 1.656)
Intermacs			
1	71 (42.3)		
2	29 (17.3)		
3	31 (18.5)		
4	37 (22.0)		

Abbreviations: LVAD, left ventricular assist device; BMI, body mass index.

 Implantation of LVAD systems with axial flow (Thoratec Heart Mate II^®^) as well as systems with centrifugal flow (Thoratec Heart Mate III^®^, HeartWare HVAD^®^ and MVAD^®^) were included. Data on pre- and postoperative AF was retrieved from medical records and pre-discharge ECG. MPE and related complications were defined as postoperative ischemia of lower or upper extremities requiring surgical intervention or visceral ischemia detected by non-invasive imaging (e.g. contrast enhanced computed tomography) necessitating surgical or pharmacological therapy (i.e. laparotomy, endovascular angioplasty and/or intravenous vasodilator therapy). Follow-up was carried out on regularly basis every three to six months during the study period. The maximum follow-up was 2282 days and medium follow-up 459 ± 518 days. Primary outcome of the study was defined as mortality.

###  Statistical analysis

 For statistical analysis, SPSS statistical package version 25.0 (IBM Corporation, Armonk, NY, USA) was used. The results are reported as mean ± standard deviation (SD). Hazard ratio for death (HR) and its corresponding asymptotic 95% confidence interval (CI) are indicated. Cox regression analysis was used to identify independent risk factors for death. Postoperative survival was estimated by using the Kaplan-Meier method and the groups were compared by log rank test. Statistical significance was considered for *P* < 0.05.

## Results

 Within the study period, a total of n = 168 patients (141 male) with end-stage heart failure underwent LVAD implantation in our institution. One hundred patients suffered from ischemic cardiomyopathy (ICM) and 68 from dilated cardiomyopathy (DCM). The mean follow-up period was 36.3 months (range: 0.1–114.5). Follow-up was complete for all surviving patients (n = 126). The mean age was 56.8 ± 11.9 years and 141 (84%) were male. The mean weight was 82.01 ± 16.6 kg (range: 38–135 kg) with a mean body mass index (BMI) of 26.7 ± 5.0. Mean ejection fraction (EF) was 16.5 ± 4.8%. Of all patients included in the analysis, 62 patients had preoperative atrial fibrillation before the LVAD implantation (Table 1). The group of preoperative MCS includes n = 74 (44.0%) patients, of which 41(24.4%) patients were supported only with extracorporeal life support (ECLS, i.e., veno-arterial ECMO), 18 (10.7%) patients with combined use of ECLS and other MCS devices, isolated Impella (n = 6; 3.6%), or IABP (n = 9; 5.4%) (Table 2).

**Table 2:** Overview of preoperative temporary mechanically support and support configurations

	**n (%)**	***P*** ** Value**	**Hazard ratio for death (95.0% CI, lower – upper)**
Preoperative mechanical circulatory support	74 (44.0)	0.160	1.391 (0.878 – 2.206)
Only ECLS (extracorporeal life support)	41 (24.4)		
Combined with ECLS/ECMO (extracorporeal membrane oxygenator)	18 (10.7)		
ECLS + Impella^®^	6 (3.6)		
ECMO + IABP	1 (0.6)		
ECLS + IABP	10 (6.0)		
ECLS + IABP + Impella^®^	1 (0.6)		
Only Impella^®^	6 (3.6)		
Only IABP	9 (5.4)		

A total of n = 74 (44 %) of patients were dependent on preoperative mechanically assistance. IABP, intra-aortic balloon pump.

Table 3 shows the operative procedures including operative techniques and concomitant surgery during LVAD implantation. Most patients were operated on-pump. The most common concomitant procedure was left atrial appendage closure.

**Table 3:** Overview of operative techniques and concomitant surgery during LVAD implantation

**Variable **	**n (%)**	***P*** ** Value **	**Hazard ratio for death (95.0% CI, lower – upper)**
Concomitant cardiac surgery	16 (9.5)	0.109	0.438 (0.160 – 1.201)
LAA (left atrial appendage) closure	4		
ASD (atrial septal defect) closure	2		
PFO (patent foramen ovale) closure	2		
Biol. aortic valve replacement (AVR)	4		
Biol. AVR and LAA closure	1		
Biol. AVR and tricuspid valve reconstruction	1		
Repeated biol. aortic valve replacement	1		
Thrombectomy of the left ventricle	1		
Surgery with HLM (heart-lung machine) via sternotomy	130 (77.4)	0.785	0.927 (0.539 – 1.594)
Surgery with peripheral ECLS via sternotomy	30 (17.9)		
Surgery without HLM (Off pump)	8 (4.8)		

Abbreviations: LVAD, left ventricular assist device; ECLS, extracorporeal life support.

###  Postoperative outcome

 A singular postoperative vascular, hemostaseological, arrhythmogenic event occurred in 107 patients (63.7%), which was significantly and independently associated with death (*P* = 0.020, HR 1.815, CI = 1.098–3.000). Twenty-seven patients (16.1%; *P* = 0.121) had vascular or visceral complications necessitating surgery. Vascular surgery intervention was necessary in 24 (14.3%) cases and visceral intervention in 7 cases (4.2%) to treat a postoperatively acquired vascular complication after LVAD implantation. Four patients (n = 2.4%) needed both kinds of surgical intervention (Table 4).

**Table 4:** Postoperative complications. Overview of observed postoperative adverse events following LVAD implantation

**Variable**	**n (%)**	***P*** ** Value**	**Hazard ratio for death (95.0% CI, lower – upper)**
Complications	107 (63.7)	0.020	1.815 (1.098 – 3.000)
Complications necessitating vascular or visceral surgery	27 (16.1)	0.121	1.587 (0.885 – 2.845)
**Vascular group**	24 (14.3)		
Thromboembolic limb ischemia	12		
Bleeding	2		
Compartment syndrome	4		
Inguinal lymphocele	2		
Wound healing disorders	2		
Other	2		
**Visceral group **			
Visceral ischemia	7 (4.2)		
Ileus	5		
Gastrointestinal bleeding	1		
**Vascular and visceral group**	4 (2.4)		
Diffuse bleeding	29 (17.3.0)	0.351	1.345 (0.721 – 2.506)
Pericardial effusion	11 (6.5)	0.602	1.249 (0.541 – 2.884)
Hemostaseological disorders	15 (8.9)	0.023	2.267 (1.119 – 4.593)
Postoperative cardiopulmonary resuscitation	13 (7.7)	0.026	2.322 (1.107 – 4.870)
Postoperative cardiac arrhythmia	53 (31.5)	0.316	1.281 (0.789 – 2.079)
Low cardiac output syndrome	28 (16.7)	0.194	1.473 (0.821 – 2.643)
Right heart failure	45 (26.8)	0.063	1.582 (0.975 – 2.565)
Stroke	8 (4.8)	0.051	2.310 (0.998 – 5.349)
Postoperative delirium	28 (16.7)	0.642	1.153 (0.632 – 2.104)
Pulmonary insufficiency	59 (35.1)	0.005	1.937 (1.219 – 3.076)
Gastrointestinal complications	23 (13.7)	0.014	2.086 (1.160 – 3.750)
Infection	50 (29.8)	0.018	1.786 (1.105 – 2.887)
Wound healing disorder	24 (14.3)	0.142	1.549 (0.864 – 2.778)
Acute kidney failure	71 (42.3)	< 0.001	2.723 (1.706 – 4.345)

Abbreviations: LVAD, left ventricular assist device; ECLS, extracorporeal life support.

 Within the vascular group (n = 24, 14.3%) thromboembolic limb ischemia occurred in 12 patients, n = 5 (41.7%) of them suffering from preoperative AF. Most likely thromboembolism was associated with ECLS or IABP explantation (Table 4). Four patients suffered leg compartment syndromes and two patients experienced inguinal lymphocele. Leg swelling, foot necrosis, wound healing disorder (n = 1) and intravascular loss of a transfemoral guide wire with the need for percutaneous explantation (n = 1) occurred as further complications. In the visceral group (n = 7; 4.2%) five patients had a visceral ischemia, one patient had an ileus and one patient had endoscopically confirmed gastrointestinal bleeding (Table 4). Postoperative right heart failure with temporary mechanical right heart assistance (RVAD) was observed in about one fourth of the patients.

###  Mortality

**Figure 1:**
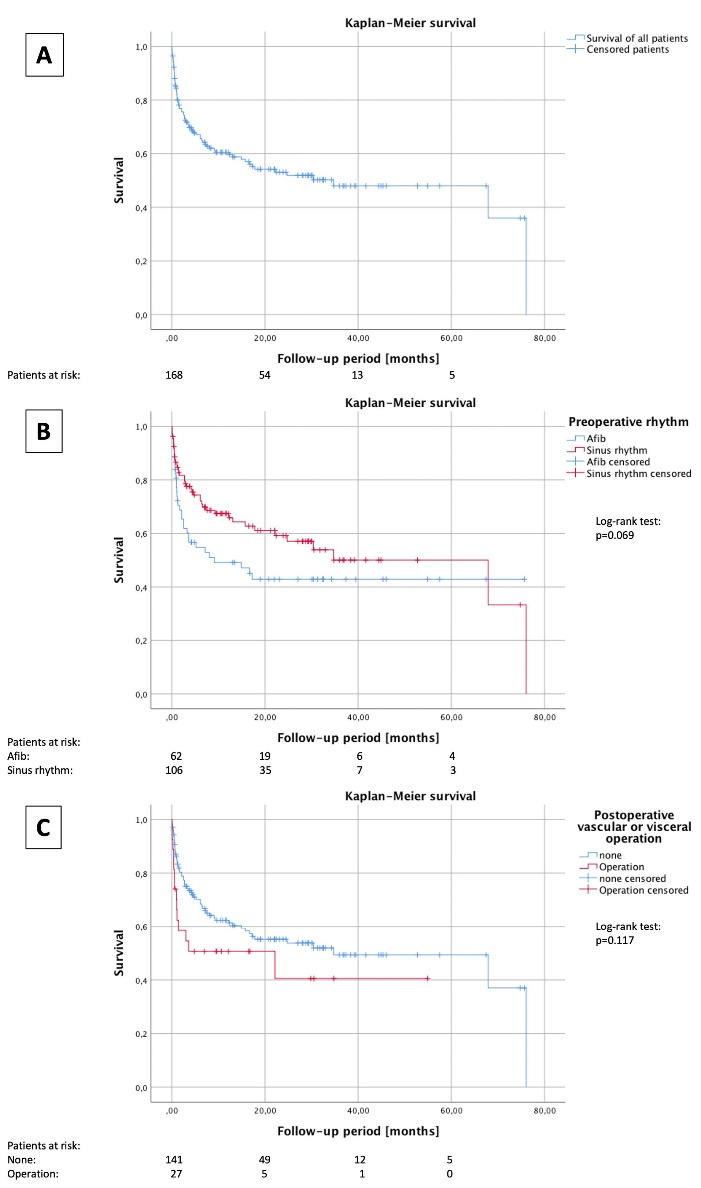
Postoperative survival. A, Kaplan-Meier survival curve of the whole cohort. Survival after LVAD implantation of all included patients (n = 168). B, survival stratified for preoperative heart rhythm. After LVAD implantation survival curve was inferior in patients with preoperative atrial fibrillation (Afib, n = 62) as compared to sinus rhythm (n = 106). C, Kaplan-Meier survival curve of regarding postoperative vascular or visceral operation. Survival after LVAD implantation of patients without (none, n = 141) and with need for postoperative vascular or visceral operation (n = 27). Patients were censored in case of heart transplantation, LVAD explantation or lost to follow-up. Afib, atrial fibrillation; LVAD, left ventricular assist device.

 The 30-day mortality rate was 16.1% (n = 26) for all patients (Figure 1A), 20.0% for the preoperative AF-group, 13.7% for the non-AF group (Figure 1B), 26.9% for the group with postoperative surgical intervention and 14.0% for the group without postoperative intervention (p = 0.117) (Figure 1C). After 12 months, the survival rates were 46.4% in the preoperative AF group and 57.7% the non-AF group (p = 0.069) (Figure 1B).

## Discussion

 LVAD implantation has evolved to a valuable and highly effective therapy for patients with end-stage heart failure. A significant proportion of patients undergo LVAD implantation in the setting of a therapy concept referred to as ‘bridge to transplantation’. Thirty percent of the patients who were listed for heart transplantation and received LVAD implantation underwent heart transplantation within the first year after the LVAD implantation.^3,15,16^

 According to INTERMACS report in 2017,^15^ overall survival has been 81% at 12 months for patients being treated with LVAD after 2008.^15^ In our study, overall survival was only 53.0% at 12 months. Therefore, we can describe a higher early mortality rate within the first year compared to the INTERMACS^15^ data, which was probably caused by the nearly three times higher percentage of INTERMACS patient profile I in our cohort (42.3% in our cohort vs. 15.3% in the INTERMACS Report). In addition, our long-term outcome was even better than global registry data.

 Risk factors for mortality were older age, female sex, critically ill patients and long-term LVAD support. The survival rate for long-term support (Kaplan-Meier 5-year survival) without heart transplantation was estimated to be 35% (INTERMACS).^15^ In our study, older age (> 65 years) and female sex were not relevant risk factors for mortality. Kaplan-Meier analysis of our cohort revealed a survival rate for long-term support to be about 50% (Figure 1). Non-cardiac comorbidities, such as chronic pulmonary disease, peripheral vascular disease, renal dysfunction, and overall nutritional state (higher body mass index, BMI) were further risk factors for mortality. According to the current literature, biventricular assist device implantation, pre-implantation dialysis, and increased surgical complexity due to previous cardiac surgery or other concomitant cardiac surgeries have been identified as major predictors for early mortality.^15^ In our statistical analysis, we found preoperative AF (*P* = 0.069) to be a potential predictor for impaired outcome after LVAD implantation. The Kaplan-Meier curve estimated a survival rate of approximately 42% at 60 months for the preoperative AF-group and 50% for the non-AF group (long rank test: *P* = 0.069). Increased surgical complexity caused by a preoperative ECLS (n = 74, *P* = 0.160) was not a relevant contributor to mortality. Neurological complications after LVAD implantation were described as relevant major adverse events, and can be either hemorrhagic or ischemic. According to the current literature^4-6^ risk factors for these events were older patient age, high blood pressure and irregular international normalized ratio (INR) levels.^17,18^ The ischemic or hemorrhagic stroke rates in the literature of 6% and 8%^18^ correspond to our results with a stroke rate of 4.8%. Further relevant major adverse events that have been reported in the literature are surgical bleeding in the early postoperative phase and gastrointestinal bleeding after the first 3 months (22%).^17,18,19^ These latter event rates are similar to our experience, with diffuse bleeding observed in 17.3% and gastrointestinal bleeding in 13.7% of our patients. Vascular events such as leg or arm ischemia after LVAD implantation were seldom, and its effects on outcome of long-term LVAD are not well described in the literature support. In a meta-analysis of three studies, Cheng^15^ described that 12 out of 53 patients suffered from leg ischemia after LVAD implantation compared to 2 out of 47 after IABP. They observed no significant difference in the incidence when comparing both methods.^20^ In our earlier studies we observed more complications such as limb ischemia (27%) and visceral ischemia (15%) after implantation of an ECLS.^5,21^ The timespan until implantation of a selective perfusion catheter for the antegrade perfusion of the superficial femoral artery has been reported to be a significant factor for increased complication rates^15^. In this study, we observed twelve cases of limb ischemia (7.1%) with only two (1.2%) resulting from a thromboembolic event. An additional eight cases were observed after ECLS implantation. In this study, we can demonstrate that preoperative ECLS was not a significant risk factor for mortality (*P* = 0.160) and the need for vascular or visceral surgery after LVAD implantation was not a relevant predictor for mortality (*P* = 0.121). Thromboembolic events with limb ischemia after LVAD implantation were very seldom and did not influence the prognosis when analyzing the overall cohort.

 Data presented in this report has been partly collected and analyzed retrospectively. Furthermore, the presented data and analysis reflect a single-center experience, with all the well-known limitations associated with a single-center study. Moreover, due to the size of our study cohort, several events with a relatively low incidence rate might be underestimated, e.g., thromboembolic events. In addition, because of the short follow-up period for the majority of patients, the known disproportionally high first-year mortality after LVAD implantation most likely underestimates the longer-term survival of the cohort that was assessed by the Kaplan-Meier method. Finally, the included patients had received different models of LVAD systems, which significantly differ in design, and may therefore also differ in their biocompatibility and their contribution to specific forms of postoperative complications. Analysis of larger cohorts of LVAD patients will allow for exploration of the herein considered questions in selective sub-cohorts.

## Conclusion

 In summary, we demonstrate that preoperative AF is a potential predictor of mortality and worse long-term outcome for patients on LVAD support. In contrast, preoperative ECLS and associated postoperative vascular adverse events did not correlate with postoperative mortality. In particular, vascular or visceral surgery as a response to these malperfusion events are not limiting factors for outcomes and had no influence on mortality.

## Acknowledgments

 We gratefully acknowledge the work of the members of the heart failure team at the Medical Faculty and University Hospital Duesseldorf.

## Funding

 None.

## Ethical approval

 The study was approval by the Local Ethics Committee 2020 - 832.

## Competing interest

 Authors declare no conflict of interests in this study.
